# The Basal Pharmacology of Palmitoylethanolamide

**DOI:** 10.3390/ijms21217942

**Published:** 2020-10-26

**Authors:** Linda Rankin, Christopher J. Fowler

**Affiliations:** Department of Integrative Medical Biology, Umeå University, SE-901 87 Umeå, Sweden; linda.rankin@umu.se

**Keywords:** palmitoylethanolamide, peroxisome proliferator-activated receptor-α, fatty acid amide hydrolase, *N*-acylethanolamine acid amidase, low back pain–sciatica, atopic eczema

## Abstract

Palmitoylethanolamide (PEA, *N*-hexadecanoylethanolamide) is an endogenous compound belonging to the family of *N*-acylethanolamines. PEA has anti-inflammatory and analgesic properties and is very well tolerated in humans. In the present article, the basal pharmacology of PEA is reviewed. In terms of its pharmacokinetic properties, most work has been undertaken upon designing formulations for its absorption and upon characterising the enzymes involved in its metabolism, but little is known about its bioavailability, tissue distribution, and excretion pathways. PEA exerts most of its biological effects in the body secondary to the activation of peroxisome proliferator-activated receptor-α (PPAR-α), but PPAR-α-independent pathways involving other receptors (Transient Receptor Potential Vanilloid 1 (TRPV1), GPR55) have also been identified. Given the potential clinical utility of PEA, not least for the treatment of pain where there is a clear need for new well-tolerated drugs, we conclude that the gaps in our knowledge, in particular those relating to the pharmacokinetic properties of the compound, need to be filled.

## 1. Introduction

**Figure 1:**
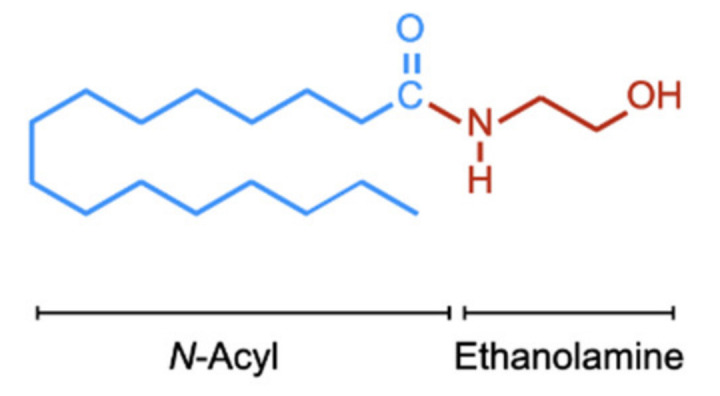
Chemical structure of PEA. For the *N*-acyl side chain, the nomenclature is (16:0) given that there are sixteen carbon atoms and no double bonds between the carbon atoms. The corresponding numbers for OEA and AEA are (18:1) and (20:4), respectively.

Palmitoylethanolamide (PEA, *N*-hexadecanoylethanolamide, structure see Figure 1) was first identified in egg yolk, soybean, and peanut oil in 1957 [1] and thereafter in mammalian tissues in 1965 [2]. It is one of the most common of the *N*-acylethanolamines (NAEs), which include the endogenous cannabinoid receptor ligand anandamide (AEA, arachidonoylethanolamide) and the satiety agent oleoylethanolamide (OEA). The original identification of PEA [1] and the demonstration in the same study that the compound was efficacious in a local passive joint anaphylaxis assay in the guinea pig was motivated by early studies suggesting that a component of egg yolk could have beneficial effects in rheumatic arthritis [3,4], and subsequent clinical studies have suggested that PEA may have a useful role to play in the treatment of a variety of afflictions ranging from pain [5] to eczema [6].

**Figure 2:**
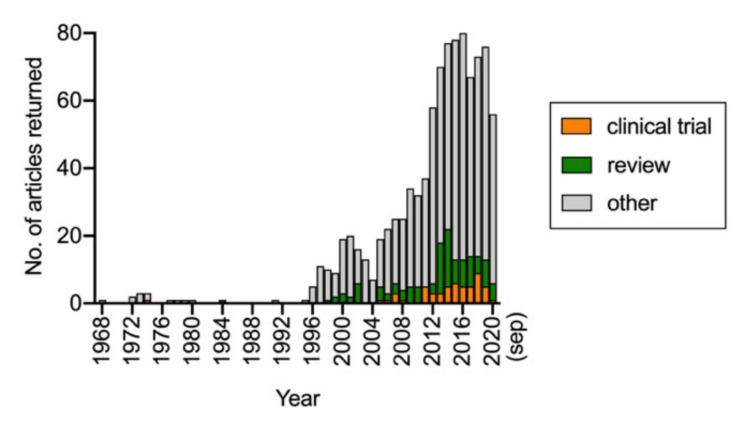
Results of a PubMed search conducted on 17 September 2020 with the search word “palmitoylethanolamide” and range 1968–present. Subsections with “clinical trial” and “review” indicated as article type were also downloaded from PubMed.

Since the early studies, there has been a steady increase in interest in PEA, and a simple PubMed search with search word “palmitoylethanolamide” indicates that since 2012, around 70–80 publications per year are concerned with this lipid (Figure 2). A breakdown of the 64 published papers (i.e., not including review articles or one corrigendum) and identified by the search over the 12-month period from September 2019 to September 2020 indicated more than half of the papers dealt either with the effects of PEA in animal or cellular models (17 articles) or levels of PEA in metabolomic/lipidomic studies of either disorders or pharmacological/nutritional interventions (19 articles). Only two studies dealing with formulations of PEA were published, and none were concerned with the pharmacokinetic properties of PEA after its absorption. In the present review, we have focused upon the ADME (absorption, distribution, metabolism, and excretion) and pharmacological targets of PEA with the aim of highlighting our current knowledge, and, as importantly, the gaps in our knowledge.

## 2. The ADME of PEA

### 2.1. Introduction

**Figure 3:**
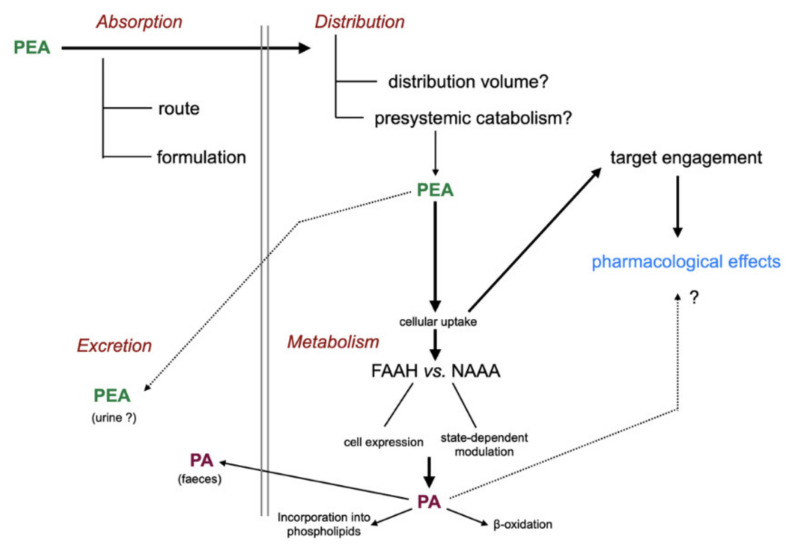
Absorption, distribution, metabolism, and excretion of PEA. Abbreviations: FAAH, fatty acid amide hydrolase; NAAA, *N*-acylethanolamine acid amidase; PA, palmitic acid. For details with respect to the metabolism of PA, see [7].

The “life cycle” of administered PEA is shown schematically in Figure 3. Briefly, after absorption (and potential presystemic metabolism), PEA is distributed into the different tissues of the body where it acts upon its pharmacological targets before being metabolised and excreted.

### 2.2. Absorption and Presystemic Metabolism of PEA

PEA is a highly lipophilic compound that raises issues concerning its formulation for optimal absorption (one of the current authors remembers the solubility of PEA being described to him as “trying to dissolve stones”). Most work upon the formulation of PEA has concerned the usefulness of micronisation, whereby the particles of PEA are made smaller, thereby presenting a larger surface area to aid the absorption (i.e., “pebbles” in the stones description above). Certainly, unmicronised, micronised, and ultra-micronised PEA formulations are absorbed following oral administration as demonstrated by measurements, for example, of efficacy in animal models (e.g., [8]) or in human or animal disorders (e.g., [5,9]). There is little data available concerning the actual absorption phase or whether the rate of absorption can be improved, although a study investigating the pharmacokinetics of a single topical ocular administration of PEA in a nanostructured lipid carrier system indicated that the compound could be detected in the rabbit retina within 50–180 min (the maximum time point) after administration [10]. In contrast, a PEA aqueous suspension did not produce detectable levels in the retina, although it was detected, at lower levels than the nanoparticle formulation, in the lens and vitreous humour [10].

In addition to its absorption, the presystemic metabolism of PEA is an important determinant of its bioavailability. The hydrolytic enzymes involved in PEA metabolism are expressed in the intestine and the liver (see Section 2.5), and upon incubation of rat liver homogenates with 50 nM PEA, a half-life of the lipid of about 25 min was found [11]. To our knowledge, there is no information in the literature about the bioavailability of PEA or, perhaps more importantly, how this varies between individuals. One way of circumventing presystemic metabolism is the use of PEA prodrugs. In this respect, an l-valine prodrug, 2-(palmitoylamino)ethyl l-valinate hydrochloride, was resistant to hydrolysis in the liver, but it released PEA via esterase catalysis in plasma samples. However, an equimolar oral dose of the compound produced lower plasma PEA concentrations than PEA itself [11], which may point to a poor bioavailability of the prodrug. An alternate approach is the use of stable PEA analogues, and palmitoylallylamide has been shown to have analgesic actions in animal models of neuropathic pain [12]. However, such compounds are no longer endogenous to the human body and thus have a considerably greater regulatory documentation requirement than PEA.

### 2.3. Distribution of PEA

As with the absorption and presystemic metabolism of PEA, data on the distribution of PEA are few and far between, and in most cases, they are confined to measures of blood levels of the compound after oral administration (see e.g., [8,11]). Thus, for example, following a dose of 300 mg of micronised PEA to humans, an approximate doubling of levels of the lipid were seen in plasma two hours after administration, falling back to normal levels by 4 and 6 h [13]. Using the data of [11], we were able to estimate the volume of distribution for a given bioavailability value and ratio of first-order absorption and elimination rate constants in the rat. Our calculations suggested that even at a low bioavailability (1%), the volume of distribution was considerably greater than the plasma volume [14]. This is admittedly not a surprising result, given the lipophilic nature of PEA, but it does raise the question as to the tissue distribution of PEA following oral administration. In this respect, Artomonov et al. [15] reported that in the rat, approximately 1% of the oral dose of [9–10-^3^H]PEA was recovered in the brain, particularly in the hypothalamus, with notable accumulation also in the pituitary and adrenal glands.

### 2.4. Cellular Uptake of PEA

The main target of PEA action, peroxisome proliferator-activated receptor-α (PPAR-α, see Section 3.1), is intracellularly located and thus requires the cellular uptake of PEA once the compound has reached the tissue. In an early study, it was demonstrated that the incubation of C1300 N18 neuroblastoma cells with [1-^14^C]PEA resulted in the labelling of cytoplasmic, microsomal and plasma membranes, with the cytoplasmic labelling remaining rather constant after 15 min of incubation (the microsomal levels increased and the plasma membrane levels decreased) [16]. Although it has thus been known for a long time that PEA can be accumulated by cells, the mechanism(s) by which this occurs is still unclear. Most work in this respect has been undertaken using the endocannabinoid homologue AEA (for reviews arguing for and against the existence of a designated plasma membrane transporter, see [17] and [18], respectively). However, what is clear is that the hydrolysis of NAEs regulate their cellular accumulation, by ensuring that the relative extra/intracellular NAE concentration is preserved [19].

### 2.5. Metabolism of PEA

In contrast to the paucity of data with respect to PEA absorption and distribution, a great deal is known concerning the metabolism of PEA. PEA is enzymatically hydrolysed to form palmitic acid and ethanolamine. The first demonstration of this was by Bachur and Udenfriend in 1966 using rat liver microsomes [20], and the enzyme involved, subsequently termed fatty acid amide hydrolase (FAAH), was characterised in detail by the Schmid group in 1985 using OEA as substrate [21]. The enzyme is a membrane-bound heterodimer localised to the endoplasmic reticulum with a pH optimum in the range of 8–9 and a wide substrate specificity encompassing *N*-acylethanolamines, *N*-acylamines, and *N*-acyltaurines [21,22,23]. A second FAAH enzyme, termed FAAH-2, has been found in humans and is localised to lipid droplets [24,25]. For both FAAH and FAAH-2, AEA is hydrolysed at a faster rate than PEA [24]. In contrast, the lysosomal enzyme *N*-acylethanolamine acid amidase (NAAA) has a pH optimum of ~5 and hydrolyses PEA much more effectively than AEA [26,27].

The existence of two different classes of PEA–hydrolytic enzymes raises the question as to which is the most important with respect to the catabolism of PEA. The short answer to this question is that it is dependent upon which tissue/cell line is under study, whether the disease process per se has affected the relative expression of FAAH and NAAA and whether we are considering endogenous or exogenous PEA. Endogenous and exogenous PEA are considered separately in the two following subsections.

#### 2.5.1. Hydrolysis of Endogenous PEA

There are now a large number of selective FAAH and NAAA inhibitors available with which to investigate the relative importance of the two enzymes with respect to PEA catabolism. Treatment with the selective FAAH inhibitor URB597 produced a large increase in PEA levels in the rat brain and liver, whereas PEA levels in the duodenum was not affected [28], and a decreased level of PEA was seen in the paw of vehicle-treated rats following an intraplantar injection of URB597 [29]. Other selective FAAH inhibitors, such as PF-3845, also increase brain and liver, but not colon PEA levels [30,31]. However, colon levels of PEA are increased in animals with experimental colon inflammation treated with the selective NAAA inhibitor AM9053 [31], and the selective NAAA inhibitor ARN077 restores the reduced PEA levels seen in the sciatic nerve following chronic constriction injury [32] and in paws treated with complete Freund’s adjuvant [33]. The different effects of FAAH vs. NAAA inhibition are presumably due to the relative expression of the two enzymes in the different tissues (not least in inflammation models given the high expression of NAAA in macrophages [27]), although in interferon-γ-treated human T84 colon carcinoma cells, PEA levels are increased to a greater extent with URB597 than with the NAAA inhibitor pentadecylamine, despite the fact that the expression at the level of mRNA of *NAAA* is slightly greater than that of *FAAH* [34]. In the mouse J774 macrophage cell line, PEA levels are increased to about double vehicle values following treatment with either PF-3845 or AM9053 [35].

#### 2.5.2. Hydrolysis of Exogenous PEA

Following the uptake of NAEs into the cell, fatty acid binding proteins and other proteins act as intracellular carriers delivering the lipids to FAAH, and in the brain, PEA, OEA, and AEA levels are increased by local administration of the fatty acid binding protein 5 inhibitor SBFI26 [36]. In contrast, the local administration of SBFI26 into the paw is without effect on the levels of these lipids [36]. Given that NAAA but not FAAH inhibition increases PEA levels in the paw [29,33], a reasonable conclusion is that fatty acid binding proteins do not deliver NAEs to lysosomal NAAA. This may be of importance with respect to the catabolism of exogenous PEA if fatty acid binding proteins play a predominant role in its intracellular transport following its uptake into the cells in question: under such conditions, FAAH would play the predominant role in its catabolism. This appears to be the case in intact human T84 colon carcinoma cells, where the hydrolysis of ≈0.1 µM PEA added to the medium is greatly reduced by URB597 (1 µM), but it is only modestly affected by the NAAA inhibitor pentadecylamine (30 µM) and not affected by the NAAA inhibitor diacylamine (10 µM) [34]. In this case, the mRNA levels for *NAAA* and *FAAH* were similar [34]. In mouse RAW264.9 macrophage cells treated for 24 h with lipopolysaccharide and interferon-γ, where mRNA levels for *Naaa* are ≈3-fold greater than for *Faah* and the added concentration of PEA was higher (10 µM, which may be important with respect to the capacity of the fatty acid binding protein carrier pathway and its selectivity relative to other potential intracellular pathways), URB597 and pentadecylamine reduce PEA hydrolysis equally [37]. Taken together, these data would suggest that the distribution and hence local concentration of PEA, as well as the relative expression of FAAH, NAAH, and intracellular carrier(s) in the cells all contribute to the catabolism of exogenously administered PEA.

### 2.6. Excretion of PEA

The metabolism of palmitic acid is well described [7], and in intact cells, the metabolic cascade PEA → palmitic acid → incorporation into phospholipids has been demonstrated [16]. To our knowledge, it is not known the extent to which orally or topically administered PEA is hydrolysed to palmitic acid prior to its excretion from the body (to say nothing about kidney function, not least because PEA itself has been reported to have a protective effect towards the kidney in spontaneously hypertensive rats [38]). Additionally, we could not find any data concerning the route of excretion of unmetabolised PEA, other than a statement (without citation or corroborating data) on an advertisement for a PEA preparation that stated excretion was renal [39]. It would be of great value if companies in possession of such information released the data to the scientific community.

## 3. PEA Targets

### 3.1. Introduction

**Figure 4:**
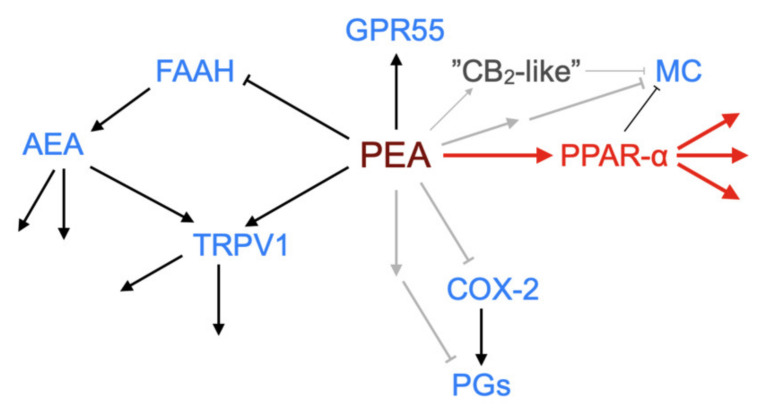
Schematic showing demonstrated and potential molecular targets of PEA. ↑, activation; ┴, inhibition (in the case of FAAH by substrate competition). The canonical pathway via PPAR-α is shown in red. Grey arrows indicate possible pathways not yet identified. Abbreviations (where not already indicated), TRPV1, Transient receptor potential vanilloid 1; MC, mast cell; COX-2, cyclooxygenase-2; PGs, prostaglandins.

One of the earliest findings with PEA was its ability to reduce the degranulation of mast cells in vivo in the ear pinna in response to substance P [40]. Since then, PEA has been shown to produce a multitude of actions in the body at the level of lipids such as the endocannabinoid 2-arachidonoylglycerol and the lipoxins 5-, 12-, 15- and 20-hydroxyeicosatetraenoic acid, and at the level of functionality in animal models of pain and inflammation (see [8,13,31,41,42,43] for examples). A key question concerns whether this multitude of effects can be ascribed to a single or to multiple primary targets (see Figure 4 for a schematic).

### 3.2. PPAR-α

PPAR-α belongs to the family of peroxisome proliferator-activated receptors, which are transcription factors in the nuclear receptor superfamily. There are a number of endogenous and synthetic PPAR-α activators, such as arachidonic acid and the fibrate family (examples include gemfibrozil, fenofibrate, and bezafibrate, which are used for the treatment of hyperlipidaemia and hypercholesterolaemia), respectively. The activation of PPAR-α results in an altered transcription of a large number of genes ranging from those coding for proteins involved in fatty acid transport and metabolism to those coding for pro-inflammatory molecules and oxidative stress [44,45]. Anti-inflammatory effects of PPAR-α agonists involve the transrepression of pro-inflammatory transcription factors such as NFκB, leading to an inhibition of the release of inflammatory cytokines such as tumour necrosis factor α (TNF-α) and interleukins 1β and 6 [44].

In 2005, Lo Verme and colleagues [46] demonstrated that PEA can activate PPAR-α. In HeLa cells transfected with a luciferase reporter gene and a plasmid containing the PPAR-α ligand binding domain, PEA produced an activation signal with an EC_50_ value of 3 µM, whereas it did not produce a signal in corresponding cell systems expressing the PPAR-β/δ or PPAR-γ ligand binding domains [46]. In vivo, PEA produced its expected effects in the carrageenan oedema model in wild-type but not PPAR-α^−/−^ mice, and a similar result was seen for the 12-O-tetra- decanoylphorbol-13-acetate ear oedema model [46], and, in a follow-up study, in the mouse formalin model of prolonged pain [47]. The follow-up study also potentially resolved an earlier dilemma, namely that in some animal models, the beneficial effects of PEA were blocked by the cannabinoid CB_2_ receptor inverse agonist SR144528, despite the fact that PEA has no direct effects at these receptors (e.g., [42]). LoVerme et al. [47] found that SR144528 blocked the effects not only of PEA in the formalin model, but also the effects of the synthetic PPAR-α agonist GW7647, and that PEA retained its effects in CB_2_^−/−^ mice. However, the compound did not block the transactivation of PPAR-α by PEA in vitro in the transfected HeLa cells, leading the authors to conclude that the effects of SR144528 are an off-target action of the compound but downstream of PPAR-α. However, there are examples where the effects of PEA are not affected by PPAR-α antagonists but are sensitive to the CB_2_ receptor inverse agonist AM630 (see below), and so some effects of PEA mediated by CB_2_ (or CB_2_-like) receptors cannot yet be ruled out, provided of course that the effect of AM630 is not an off-target action upon another receptor [48].

PPAR-α has been implicated as the prime mediator of PEA in a variety of different animal models (Table 1; note that almost all the studies are undertaken on males alone. Note also that in some of the cases, authors show the “difference of the significance” between, for example, effects in PPAR-α^+/+^ mice and PPAR-α^−/−^ mice as opposed to the “significance of the difference” [49], which is a potentially important caveat). Given this, it would be expected that the pattern of clinical effects of PEA should match those of the fibrates. While it is true that anti-inflammatory effects of fibrates are seen in experimental models [50,51,52], PEA and fibrates do differ with respect to their unwanted effects profile. Thus, at the level of common (>1:100) unwanted effects, PEA is well tolerated (see [14]), whereas fenofibrate has a range of unwanted gastrointestinal effects including abdominal pains [53]. PEA given topically has been found to reduce the need for glucocorticoid treatment in a large cohort of patients with atopic eczema [6], whilst eczema is reported as a common unwanted effect for gemfibrozil (although admittedly, this is not as common with enofibrate and bezafibrate, and so it may be an off-target effect) [54]. In several in vivo models, the effects of PEA are not blocked by PPAR-α antagonists, suggesting that there are PPAR-α-independent effects of this compound. Thus, for example, Okine et al. [55] reported that the reduction in formalin-evoked nociceptive behaviour produced by a microinjection of PEA into the anterior cingulate cortex was not blocked by GW6471, but it was blocked by the CB_1_ receptor inverse agonist AM251. In the study of Vaia et al. [56] (notably the only one undertaken using female mice) reported in Table 1, the effect of PEA on ear swelling induced by 2,4-dinitrofluorobenzene was not antagonised by GW6471, but it was blocked by the CB_2_ receptor inverse agonist AM630. Finally, the sensitisation of HaCaT keratinocyte cells with poly-(I:C) results in expression of the chemokine monocyte chemotactic protein-2 (MCP-2). This expression is reduced by PEA in a manner that is not antagonised by the PPAR-α antagonist MK866 (or by SR144528), but it was blocked by iodo-resiniferatoxin [57]. Taken together, these observations indicate that PEA may produce at least some of its effects via additional targets that are not shared with the fibrates. Some potential targets are discussed in Section 3.3, Section 3.4, Section 3.5 and Section 3.6 below.

### 3.3. NAE Turnover

An obvious candidate for additional targets for PEA would be the hydrolytic enzyme FAAH. In this scenario, the high local concentration of PEA competes with the endogenous NAEs at this enzyme, thereby preventing their hydrolysis and increasing their levels. Certainly, the inhibition of FAAH produces anti-inflammatory effects in animal models [74], and increased AEA levels are seen in the plasma (but not in the spleen where there is a decrease) of mice after i.p. PEA treatment [41]. However, in humans and in dogs, oral PEA treatment does not produce a significant change in plasma AEA levels [13]. Transcriptional effects should also be considered: the treatment of MCF-7 human breast cancer cells for 4 days with PEA reduces the mRNA expression of *FAAH* and the ability of intact cells to hydrolyse AEA [75]. In contrast, short-term incubation (15 min) with PEA does not affect *Faah* or *Naaa* expression in rat RBL-2H3 cells [76]. Given that in contrast to PEA [5], FAAH inhibitors have failed in clinical trials as analgesics [77,78], the suggestion that the clinical efficacy of PEA (see below) is primarily via modulation of the levels of other NAEs lacks support.

**Figure 5:**
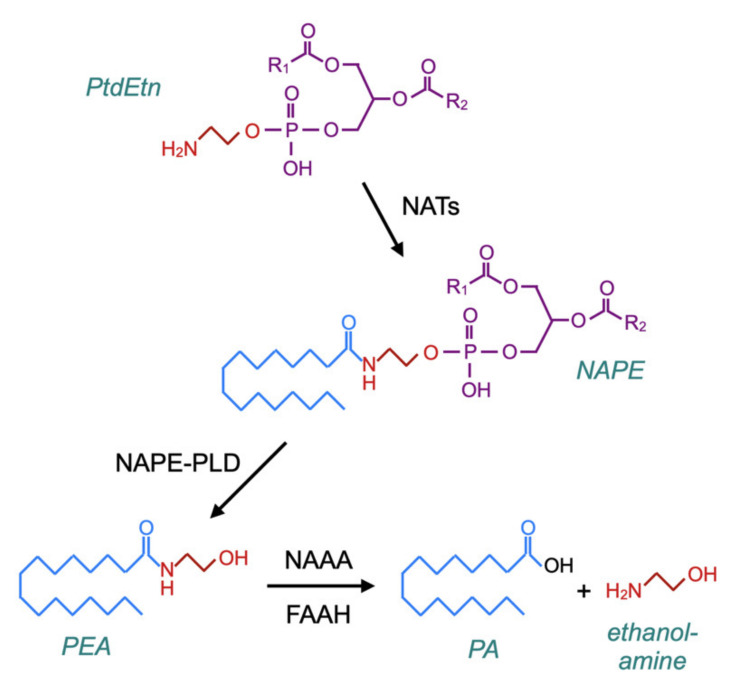
The canonical pathway for the synthesis of PEA via *N*-acyltransferases (NATs) and *N*-acylphosphatidylethanolamines (NAPE)–phospholipase D. The catabolism of PEA is also shown in the figure, which is based upon Figure 2 of [84] and Figure 1 of [86].

A related question is whether PEA administration produces feedback effects upon NAE synthesis, since such effects could in theory be detrimental to patients upon the discontinuation of treatment with the compound. The canonical pathway for the synthesis of PEA (and NAEs) was characterised by Schmid and colleagues in the late 1970s–early 1980s [79,80,81]. In brief, membrane phosphatidylethanolamine (PtdEtn)-containing phospholipids are transacylated by a calcium-dependent *N*-acyltransferase (NAT) to form *N*-acylphosphatidylethanolamines (NAPEs), which in turn are hydrolysed by NAPE-hydrolysing phospholipase D (NAPE-PLD) to form the NAEs (Figure 5). However, in mice, the genetic deletion of NAPE-PLD reduces PEA and stearoylethanolamine levels in the brain by about 40%, rather than completely preventing their formation [82], and the incubation of brain homogenates from NAPE-PLD-deficient mice with *N*-palmitoylethanolamine plasmalogen results in the formation of PEA [83] (for further information with respect to canonical and alternative pathways for NAE synthesis, see [84,85]).

**Figure 6:**
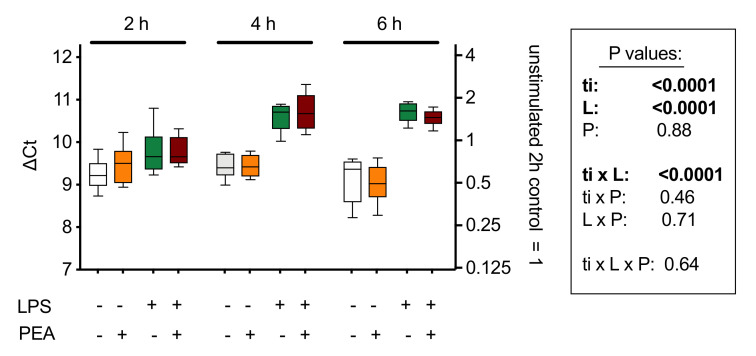
mRNA levels of *Napepld*, coding for NAPE-hydrolysing phospholipase D (NAPE-PLD) in J774 cells cultured in 24-well plates for 24 h and treated for 2, 4, and 6 h with lipopolysaccharide (LPS, 0.1 µg/mL) + interferon-γ (100 U/mL) and/or 10 µM PEA. Shown are box and whisker plots, N = 8. The mRNA values are determined using *Rpl19* as reference gene and are given as ∆Ct with the mean values relative to the unstimulated controls at the 2 h time point on the right *y*-axis. A decrease of 1 ∆Ct unit represents a doubling in the mRNA concentration. The box shows the results of a 3-way ANOVA (ti, time; L, LPS + interferon-γ; P, PEA. These indicate a time-dependent effect of the inflammatory stimulus on *Napepld* expression, but no significant effect of PEA treatment. Figure redrawn from [87].

With respect to potential feedback effects on PEA synthesis, the evidence at present suggests that this is not the case: three days of oral PEA administration to mice treated intra-colonically either with vehicle or with 2,4-dinitrobenzenesulfonic acid (to induce a colonic inflammation) showed colon levels of AEA and OEA that were not significantly different from the corresponding animals not given PEA [60]. A shorter (2–6 h) in vitro treatment of J774 macrophages with PEA also showed no changes in the mRNA expression of *Napepld* [87] (Figure 6). *Napepld* levels in RB2H3 basophilic leukaemia cells are also not significantly affected by 15 min of treatment with PEA, although AEA levels are increased [76], which might implicate effects of PEA upon non-canonical synthetic pathways (reviewed in [84,85]).

A final consideration concerns whether the beneficial effects of PEA are in fact mediated or alternatively mitigated by its hydrolysis product, palmitic acid. Certainly, palmitic acid is not without biological effects, including an ability to affect Toll-like receptor signalling involved in macrophage activation in response to lipopolysaccharide [88]. Palmitic acid can inhibit PPAR-α transactivation [89], albeit with a lower potency than PEA. However, if palmitic acid was responsible for the effects of PEA, then a blockade of PEA hydrolysis would be expected to reduce the observed actions of PEA. Our in vitro [37] study on the effect of PEA upon prostaglandin production did not see such a reduction. In a 2,4,6-trinitrobenzenesulfonic acid model of colitis, the FAAH inhibitor PF-3845 did not change the effect of PEA upon TNF-α production in the colon, but it did negate the effect of PEA upon the colon weight/length ratio [31]. Whether or not this latter effect is due to the net effect of opposing actions of the two compounds or a true blockade requires further study.

### 3.4. Transient Receptor Potential Vanilloid 1 (TRPV1) Receptors

Transient Receptor Potential Vanilloid 1 (TRPV1) receptors are receptors that respond to heat, but also to chemical agents such as capsaicin (found in chili peppers and responsible for their burning sensation), resiniferatoxin, and AEA. In 2001, Petrocellis et al. [90] demonstrated that in human embryonic kidney 293 cells transfected with TRPV1 receptors, PEA at a concentration of 5 µM (i.e., the same range as seen for its effects upon PPAR-α) potentiated the ability of AEA to activate TRPV1-mediated calcium influx by reducing its EC_50_ value from 0.44 to 0.22 µM. PEA also enhanced the responses to capsaicin and resiniferatoxin and in cell-free assays increased the potency of AEA as an inhibitor of [^3^H]resiniferatoxin binding. A subsequent study using the same cells reported a small effect per se of PEA upon calcium influx that was not seen with other unsaturated NAEs [91], and in differentiated F11 dorsal root ganglion x neuroblastoma hybrid cells naturally expressing TRPV1 receptors, PEA elicits calcium transients (EC_50_ 3 µM) in a manner reduced (but not blocked) by the TRPV1 antagonists capsazepine and SB-366791 (the latter at a concentration that completely blocked the response to capsaicin) [92]. Interestingly, these authors found that the PPAR-α antagonist GW-6471 (but not SR-144528, q.v. its purported effects upon PPAR-α signalling discussed in Section 3.2) also reduced the calcium transient response to PEA, but not to capsaicin. A similar result was seen in Chinese hamster ovary cells expressing TRPV1 receptors, although in this case, the response to PEA was totally blocked by capsaicin [92]. Taken together, these data suggest that in these cells, the effects of PEA upon calcium transients can be mediated both by direct actions upon TRPV1 receptors but also secondary to the activation of PPAR-α.

In vivo, studies have demonstrated the involvement of TRPV1 receptors in the actions of PEA. Thus, for example, a reduction of PEA responses by has been reported in the chronic constriction injury model of neuropathic pain in mice [61], whilst iodo-resiniferatoxin potentiates the effect of PEA on upper gastrointestinal transit in mustard oil-treated mice [93]. PEA given as a continuous infusion to rats reduces the vasopressor response to electrical stimulation of the thoracic sympathetic nerves in a manner partially blocked by capsazepin [94], whilst iodo-resiniferatoxin affects the actions of PEA given into the periaqueductal grey of rats upon the spontaneous firing of cells in the rostral ventromedial medulla [95].

### 3.5. GPR55 and GPR119 Orphan Receptors

In 2007, Rydberg et al. [96] reported that PEA stimulated GTPγS binding in human embryonic kidney 293 cells transfected with the orphan receptor GPR55. The ligand selectivity of this receptor has been something of a bone of contention [97], but the effect of PEA upon the ability of bone marrow-derived mouse macrophages to phagocytose fluorescent beads or apoptotic cells was not seen when the corresponding macrophages from GPR55^−/−^ mice were used [98]. PEA also causes insulin release from wild-type rat pancreatic BRIN-BD11 cells; this is not seen in the corresponding GPR55^−/−^ cells [99]. In vivo, PEA reduces myeloperoxidase activity (a neutrophil marker) in colonic tissues from mice treated with 2,4,6-dinitrobenzenesulfonic acid in a manner blocked by the GPR55 antagonist ML-191 [60] and there is evidence (dependent upon the selectivity of the inverse agonist at the dose used) that the vasodepressor effects of PEA given as a continuous intravenous infusion involves this receptor [94]. Less is known about the interaction between PEA and GPR119 [100] but with respect to the modulation of glucagon-like peptide secretion from intestinal l-cells, OEA rather than PEA is the primary NAE involved [101].

### 3.6. Downstream Effects of PEA

The focus above has been mainly concerned with target molecules, rather than the downstream effects observed following target engagement. Given the myriad changes in the body produced by the activation of PPAR-α [44,45], a detailed description of the downstream effects of PEA in the different animal models listed in Table 1 is outside the scope of the present review. However, an example will suffice to illustrate the multitude of downstream effects of PEA. Ye et al. [73] investigated the effects of PEA in a mouse model of oxygen-induced retinopathy, whereby mice were exposed to 75% oxygen for 5 days on post-natal days 7 to 12, after which time they were returned to a normoxic environment and treated with PEA for 5–15 days depending upon the experiment. The PEA treatment reduced levels at protein and mRNA levels of the angiogenic marker vascular endothelial growth factor (VEGF) and the inflammatory cytokine TNF-α, the number of TUNEL-positive cells, the avascular area as well as markers of extracellular matrix, profibrotic changes, and gliosis [73].

At a deceptively simpler level, the effects of PEA on mast cell (MC) function can be considered. Aloe et al. [40] reported that PEA (then called LG 2110/1) at a dose of 20 mg/kg s.c. reduced MC degranulation in the ear pinna in response to local administration of substance P by 35% as compared to 9% for saline. Interestingly, a shorter chain NAE, *N*-butanoylethanolamine (termed LG 2130/2 in the paper) was more potent, a dose of 1 mg/kg s.c. producing a 67% reduction of MC degranulation—this difference is greater than can be accounted for by the difference in the molecular weights of the two compounds (131 vs. 299 Da). Whether or not the difference in potency reflects solubility issues, the bioavailability at the site or action or a difference in potencies at the target molecules mediating the effects awaits elucidation. This effect of PEA is also seen in chronic granulomatous inflammation produced by the implantation of λ-carrageenan-containing sponges onto the backs of rats. The granulomatous inflammation is MC-driven, and PEA treatment both reduces mast cell-derived nerve growth factor release and the neurogenesis of sensory nerves [102]. In the dorsal root ganglia, PEA also reduced the levels of nerve growth factor, TNF-α, and cyclooxygenase-2 produced by the λ-carrageenan treatment. In a subsequent study [103], the authors demonstrated that mast cell protease-5 expression was also decreased. Whilst clearly indicating that PEA affects MC function, they do not provide information as to the underlying mechanisms whereby this occurs. In vivo treatment of 2,4-dinitrofluorobenzene-treated mice (locally on the abdomen) with contact allergic dermatitis increases the number of MC in a manner reduced by PEA, and this effect is negated by concomitant treatment with the CB_2_ receptor inverse agonist AM630, but not by the PPAR-α antagonist GW6471 [56]. There is mechanistic data in a recent paper using RBL-2H3 cells showing sensitivity to AM630 [76], but these cells resemble mucosal rather than serosal MC, and in addition give variable results both with respect to the effects of PEA [76,104,105,106] and indeed to the ability of compounds such as Substance P to produce a degranulation [76,107]. Skin cultures may be more useful to investigate mechanisms of action, and PEA has been shown inhibit compound 48/80-induced regulation of MC in organ cultures from skin obtained from dogs undergoing mastectomy [108].

## 4. The Clinical Utility of PEA

The aim of the present review has been to discuss the basal pharmacology of PEA, and so this subject is only dealt with briefly. Animal data indicate that micronised PEA has no overt toxicity even at high doses (1000 mg/kg/day p.o. for 90 days in rats [109]), and clinical trials have reported that the compound is very well tolerated—indeed, a conspicuous lack of adverse effects is a common finding in most (but not all, see below) clinical studies with PEA. Using the “rule of three” [110] and the available data at the time (2016), we calculated that the number of patients needed for a 95% likelihood of observing a single adverse drug reaction was at a frequency of <1/500 for short-term (3 weeks) PEA treatment and <1/50 (due to the smaller number of patients investigated) for longer (3 months) treatment) [14].

Early clinical trials with PEA suggested that the compound reduced the incidence of acute respiratory infections in soldiers [111]. With respect to PEA and pain (reviewed in [14]), the largest study so far published was a multi-centre, double-blind randomised study on three groups (placebo, 300 and 600 mg micronised PEA, total 636 patients, treatment duration 3 weeks) of patients with low back pain/sciatica [5], where PEA was found to be efficacious and extremely well tolerated. The article was written in a somewhat niche Spanish language journal, but the original material has been re-analysed [112], where the authors reported a Number Needed to Treat (NNT) for ≥50% pain relief of 9 (95% CI 5–29) for 300 mg of PEA and 1.7 (95% CI 1.4–2) for 600 mg. This last value is remarkably low—much lower than the drugs commonly used, which have NNT values ranging from 3.5 for tricyclic antidepressants to 7.7 for pregabalin [112]. The NNT for cannabis and cannabinoid preparations for a 30% reduction of pain for patients with chronic non-cancer pain (an admittedly wider range of conditions) was found in a meta-analysis to be 24 (95% CI 15–61), and the number of patients reporting a 50% reduction in pain was not significantly different from placebo [113]. Pain is a very heterogeneous family of disorders, and so it would be expected that the efficacy of PEA will depend upon the type of pain studied. This appears to be the case: Steels et al. [114] reported beneficial effects of PEA at 300 and 600 mg/day in a double-blind randomised placebo controlled study comprising 110 patients with knee osteoarthritis upon the primary outcome measure (Western Ontario and McMaster Universities Osteoarthritis Index (WOMAC)) and its pain sub-domain score after 8 weeks of treatment, with no adverse effects being reported by the patients. In contrast, a randomised, double-blind, placebo-controlled, parallel multi-center 12-week treatment study with 600 mg of ultra-micronised PEA as an add-on therapy in 73 patients with spinal cord injury neuropathic pain failed to find a significant difference from placebo in the primary outcome measure (change in pain intensity from −1 week baseline to the end of the study using a 10-point numeric rating scale) and in addition reported serious adverse events in both PEA and placebo groups [115].

In addition to pain, PEA has been reported to have potentially beneficial effects in a wide variety of conditions, ranging from depression (as an add-on to citalopram) [116] to systemic endothelial dysfunction in ocular hypertension [117]. In this respect, the largest study to date is that of Eberlein et al. [6], who investigated the effects of a skin cream containing PEA upon symptoms of atopic eczema in 2456 patients at 525 centres. The patients were assessed at the beginning and end (4 to 6 weeks) of the treatment. Dramatic reductions in the clinical signs and symptoms (dryness, excoriation, lichenification, scaling, erythema, pruritus) were noted, and this was also seen in patient assessments and their use of topical corticosteroids [6]. A placebo comparator was not used in this study. Most other studies are rather small in size and have not yet been confirmed in large randomised placebo-controlled double-blind clinical trials, but they do suggest that PEA may be useful for a number of disorders, not just pain.

## 5. Natural Sources of PEA

As pointed out in the introduction, the original identification of PEA was motivated by studies showing that a component of egg yolk could have beneficial effects in rheumatic arthritis [1,3,4]. PEA is found in a large number of food sources at levels ranging from 950 µg/g fresh weight in soy lecithin to 7.2 µg/g fresh weight in roasted coffee, 0.14 µg/g fresh weight in black eyed peas, and less than 10 ng/g dry weight in apples, lentils, and potatoes [118]. It is also found in human milk, where levels range from ≈0.1 to ≈3 nM [119]. To put these data in perspective, PEA in clinical trials has been used at doses in the range of 300 to 1200 mg per day. Assuming for the sake of argument that the bioavailability of PEA in food sources and in the PEA formulations used is the same, 300–1200 mg of PEA/day would require the daily consumption of 0.32–1.3 kg of soy lecithin, 42–170 kg of roasted coffee, and 2200–8700 kg of black-eyed peas. The authors suspect that the safety profile of such regimes would be less satisfactory than that seen with the PEA formulations.

## 6. Conclusions

In the present review, the basal pharmacology of PEA has been discussed, with the stated aim of identifying both the state of the art in the field but also highlighting important gaps in our knowledge. The review has been restricted to PEA per se, but there are preclinical and/or clinical studies investigating PEA in common with other agents, such as luteoline, polydatin, α-lipoic acid, and transpolydatin, where the pharmacokinetic and pharmacodynamic properties of PEA might (or might not) be different. Finally, we have not touched upon veterinary uses of PEA, restricting the short clinical section to humans.

With respect to the section on the ADME of PEA, there are large gaps in our knowledge. The bioavailability and tissue distribution of PEA is not known, and there are no published data on the route or rate of elimination of PEA. PEA is very well tolerated, but, other than in studies where it is given as an add-on treatment, interaction data with other drugs is lacking. It would be extremely useful to the scientific community if the companies with registered PEA products release any such data that they have to the public domain. With respect to the pharmacodynamics, much is now known as to how PEA produces a myriad of effects in the body. However, it is also clear that the common description in the literature of PEA as a “PPAR-α agonist” is slightly misleading in that (a) there are effects of PEA reported that do not involve this receptor and (b) that TRPV1, GPR55, and possibly CB_2_ or CB_2_-like receptor-mediated biological effects have also been reported. Additionally, we exhort journal reviewers to clamp down on descriptions of PEA, often in article titles, as an “endocannabinoid”, given that it does not interact directly with CB receptors.

Finally, with respect to the short section on the clinical utility of PEA, a wide range of potential indications have been (and need further to be) explored. In our review from 2016 on the usefulness of PEA as a treatment for pain [14], we concluded that “the available clinical data support the contention that PEA has analgesic actions and motivate the further study of this compound, particularly with respect to head-to-head comparisons of unmicronised vs. micronised formulations of PEA and comparisons with currently recommended treatments.” To our knowledge, such studies, which would without doubt increase the interest of the scientific and medical community for this versatile “pebble” that is PEA, have still not been reported in the literature.

## Figures and Tables

**Table 1:** Effects of palmitoylethanolamide (PEA) in relation to peroxisome proliferator-activated receptor-α (PPAR-α) involvement in vivo (R = rat, M = mouse).

Reference	Model	Species Strain Genus			Wt (g)	PEA Dose	PPAR-α Involvement
Aldossary et al. [58]	Inflammatory pain. Complete Freund’s adjuvant hind paw injections, Von Frey paw withdrawal	R	S-D ^a^	Male	180–250	50 µg i.pl.	Effect mimicked by WY12643 and reduced by GW6471
Alsalem et al. [59]	Osteoarthritis. Monosodium iodoacetate (MIA) in knee joint. Von Frey paw withdrawal	R	S-D	Male	180–250	50 µg Intra-articular injection	GW6471 reversed anti-nociceptive effects of PEA
Borrelli et al. [60]	2,4-dinitrobenzene-sulfonic acid induced colitis	M	ICR	Male	25–30	0.1–10 mg⋅kg^−1^ i.p or p.o	GW6471 reversed anti-inflammatory effects of PEA (as did GPR55 and CB_1_ antagonists)
Costa et al. [61]	Neuropathic pain. Chronic constriction injury of sciatic nerve.Thermal hyperalgesia	M	C57BL/6J	Male	25–30	10 mg/kg i.p.	GW6471 reversed PEA-induced anti-hyperalgesia (as did antagonists for CB_1_, PPAR-γ and TRPV1)
D’Agostino et al. [62]	Carrageenan-induced paw oedema	M	Swiss	Male	20–25	0.01–1 µg i.c.v	Effect mimicked by GW7647
D’Agostino et al. [63]	Carrageenan-induced paw hyperalgesia. Paw withdrawal.	M	Swiss	Male	20–25	0.1–1 µg i.c.v	Effect mimicked by GW7647
Di Cesare Mannelli et al. [64]	Peripheral neuropathy. Chronic constriction injury of sciatic nerve; mechanical allodynia and hyperalgesia	M	B6.129S4-SvJae-P paratm1Gonz	Male	-	30 mg⋅kg^−1^ –0.3 mL s.c.	PPAR-α^−/−^ mice
Di Paola et al. [65]	Inflammation after renal ischaemia–reperfusion injury	M	CD1	-	25–30	10 mg/kg i.p.	PPAR-α^−/−^ mice.
Di Paola et al. [66]	Model of myocardial ischemia reperfusion injury	R	Wistar	Male	250–300	10 mg/kg i.p.	PPAR-α^−/−^ mice
Donvito et al. [67]	Paclitaxel-induced allodynia	M	ICR	Male	18–35	30 mg/kg i.p.	Antagonism by GW6471
Esposito et al. [68]	Inflammatory model of Parkinson’s disease	M	-	Male	20–27	10 mg/kg, i.p	PPAR-α^−/−^ mice
Esposito et al. [69]	Dextran sodium sulphate-induced colitis	M	CD1	Male	6 weeks old	2, 10 or 50 mg/kg i.p.	Antagonism by MK866
Impellizzeri et al. [70]	Streptozotocin-induced diabetic peripheral neuropathy	M	CD1	Male	18–22	10 mg/kg i.p.	PPAR-α^−/−^ mice
Lo Verme et al. [46]	Carrageenan-induced paw oedema and phorbol ester-induced ear oedema	M	C57BL6	Male	25–30 g	10 mg/kg i.p	PPAR-α^−/−^ mice Also mimicked by PPAR-α agonists OEA, GW7647, and Wy-14643
LoVerme et al. [47]	Sciatic nerve ligation, arthritis induced by Freund’s adjuvant, Carrageenan-induced paw oedema	M + R	Swiss mice and S-D rats	Male	-	20 mg/kg s.c., 50 µg i.pl or 30 mg/kg i.p.	PPAR-α^−/−^ mice Mimicked by GW7647
Paterniti et al. [71]	Spinal cord injury (SCI)	M	-	-	20–27	10 mg/kg i.p.	PPAR-α^−/−^ mice. Also involvement of PPARs -δ and -γ
Sarnelli et al. [72]	Dextran sodium sulphate-induced colitis. Inflammation-associated angiogenesis	M	CD1	Male	-	2 and 10 mg/kg	PPAR-α^−/−^ mice
Vaia et al. [55]	Model of contact allergic dermatitis	M	C57BL/6J	Female	25–30	5 mg/kg i.p.	Ear scratches but not ear thickness was reduced by GW6471
Ye et al. [73]	Pathological neovascularisation and fibrosis in oxygen induced retinopathy model	M	C57BL/6J	-	-	30 mg/kg i.p.	PPAR-α^−/−^ mice

^a^ Sprague–Dawley.

## Abbreviations

ADME	absorption, distribution, metabolism and excretion
AEA	anandamide, arachidonoylethanolamide
COX-2	cyclooxygenase-2
FAAH	fatty acid amide hydrolase
MC	mast cell
NAAA	*N*-acylethanolamine acid amidase
NAE	*N*-acylethanolamine
NAPE	*N*-acylphosphatidylethanolamines
NAPE–PLD	NAP hydrolysing phospholipase D
NAT	*N*-acyltransferase
NNT	Number Needed to Treat
OEA	oleoylethanolamide
PA	palmitic acid
PEA	palmitoylethanolamide, *N*-hexadecanoylethanolamide
PG	prostaglandin
PPAR-α	peroxisome proliferator-activated receptor-α
PtdEtn	phosphatidylethanolamine
TNF-α	tumour necrosis factor-α
TRPV1	transient receptor potential vanilloid 1
WOMAC	Western Ontario and McMaster Universities Osteoarthritis Index
